# Ocular vestibular evoked myogenic potential (VEMP) reveals mesencephalic HTLV-1-associated neurological disease

**DOI:** 10.1371/journal.pone.0217327

**Published:** 2019-12-27

**Authors:** Tatiana Rocha Silva, Ludimila Labanca, Júlia Fonseca de Morais Caporali, Marco Aurélio Rocha Santos, Luciana Macedo de Resende, Rafael Teixeira Scoralick Dias, Denise Utsch Gonçalves

**Affiliations:** 1 Programa de Pós-Graduação em Infectologia e Medicina Tropical, Faculdade de Medicina da Universidade Federal de Minas Gerais, Belo Horizonte, Minas Gerais, Brazil; 2 Programa de Pós-Graduação em Ciências Fonoaudiológicas, Faculdade de Medicina da Universidade Federal de Minas Gerais, Belo Horizonte, Minas Gerais, Brazil; Baskent University, TURKEY

## Abstract

**Purpose:**

Vestibular Myogenic Evoked Potential (VEMP) evaluates vestibulo-ocular and vestibulo-collic reflexes involved in the function of the otolithic organs and their afferent pathways. We compared the results of cervical and ocular VEMP in HTLV-1 associated myelopathy (HAM) and HTLV-1-asymptomatic infection.

**Participants and methods:**

This cross-sectional study included 52 HTLV-1-infected individuals (26 HAM and 26 asymptomatic carriers) and 26 seronegative controls. The groups were similar regarding age and gender. Participants underwent simultaneous ocular and cervical VEMP. The stimulus to generate VEMP was a low-frequency tone burst sound tone burst, with an intensity of 120 decibels normalized hearing level, bandpass filter from 10 to 1,500 Hertz (Hz), with 100 stimuli at 500 Hz and 50 milliseconds recording time. The latencies of the electrophysiological waves P13 and N23 for cervical VEMP and N10 and P15 waves for ocular VEMP were compared among the groups. The absence or delay of the electrophysiological waves were considered abnormal results.

**Results:**

Ocular VEMP was similar among the groups for N10 (p = 0.375) and different for P15 (p≤0.001). Cervical VEMP was different for P13 (p = 0.001) and N23 (p = 0.003). About ocular VEMP, in the HTLV-1-asymptomatic group, normal waves were found in 23(88.5%) individuals; in HAM group, normal waves were found in 7(26.9%). About cervical VEMP, 18(69.2%) asymptomatic carriers presented normal waves and only 3(11.5%) patients with HAM presented normal waves. Abnormalities in both VEMPs were found in 1(3.8%) asymptomatic carrier and in 16(61.5%) patients with HAM.

**Conclusion:**

Neurological impairment in HAM was not restricted to the spinal cord. The mesencephalic connections, tested by ocular VEMP, have been also altered. Damage of the oculomotor system, responsible for eye stabilization during head and body movements, may explain why dizziness is such a frequent complaint in HAM.

## Introduction

The Human T-cell lymphotropic virus type 1 (HTLV-1) infection affects approximately 5–10 million people worldwide [1]. The majority of the infected individuals remain asymptomatic throughout their lives [2]. The host genetic and immunological factors seem to be related to the development of HTLV-1-associated diseases [1,2].

The range of neurological manifestations of HTLV-1-associated myelopathy (HAM) includes not only the spine, with the classical motor limitations affecting the lower limbs, but also the autonomic dysfunction [3]. In fact, inflammatory alterations due to HAM can be detected in the cortex, subcortical white matter, cerebellum, and brainstem, mainly in the advanced phases of this disease [4–7].

The complaint of dizziness has proven to be frequent in patients with HAM and can be one of the first symptoms of HTLV-1-neurological impairment [8,9]. Therefore, individuals infected with HTLV-1 may present vague complaints, with no motor, sensitive, or autonomic abnormalities [4–6]. HAM diagnosis is based on clinical criteria that reveals established neurological damage [10].

Vestibular Evoked Myogenic Potential (VEMP) has been established as a reliable and practical physiological test of the otolithic organs saccule and utricle and their pathways [11]. A normal VEMP depends on the functional integrity of the saccular and utricular maculae, the inferior vestibular nerve, the superior vestibular nerve, the vestibular nuclei, the central vestibular pathways, and the neuromuscular plaques involved in these reflexes [12–15]. Thus, VEMP tests the peripheral and central vestibular pathway, including the brainstem [11,12] and the vestibular reflexes such as the vestibulo-ocular, the vestibulo-collic and the vestibulospinal [11–14].

The subclinical spinal cord injury related to HAM has been already shown through VEMP of cervical and of lower limbs muscles, exams that are used to test the vestibulo-collic reflex [8,9,13,16,17]. The present study proposes the use of VEMP of the oculomotor system (ocular VEMP) to test the brainstem pathways associated with body balance to verify the extension of the HTLV-1-neurological damage.

## Methods

### Study design

The study was a comparative cross-sectional analysis. Cervical VEMP and ocular VEMP were compared between individuals with definite HAM, HTLV-1-asymptomatic carriers and healthy controls.

### Ethical aspects

This research was conducted in accordance with the principles expressed in the Declaration of Helsinki and was approved by the Research Ethics Committee from Universidade Federal de Minas Gerais (COEP UFMG), logged under protocol number CAAE 92928518.3.0000.5149. All participants provided voluntary written consent and declared that they were aware of the study procedures and their choice to participate. The written informed consent (as described in the PLOS consent form) was obtained for image publication.

### Sample size

The sample size was calculated using G* Power software 3.1.9.2 (Heinrich-Heine Universitat Düsseldorf, Düsseldorf, Germany, 2007) to achieve a power of 80% and a significance level of 5% based on the mean and standard deviation of the P13-N23 response of patients with HAM and healthy controls [8]. The final calculation included 26 participants per group.

### Participants

The groups of study were recruited from a cohort of former blood donors infected with HTLV-1 who have received follow-up from the Interdisciplinary HTLV Research Group (GIPH) since 1997, in Belo Horizonte, Brazil [18]. The GIPH evaluates the natural history, clinical manifestations and epidemiological aspects of HTLV infection.

Seventy-eight individuals, 32 to 60 years of age, were invited to participate in this study. The participants consisted of 26 individuals with definite HAM, 26 with HTLV-1-asymptomatic infection, and a control group of 26 individuals not infected by HTLV-1. The control group consisted of the active blood donors followed by GIPH as the negative controls.

The classification of the participants infected by HTLV-1 regarding neurological impairment followed the Expanded Disability Status Scale (EDSS) [19] and the Osame´s Motor Disability Score (OMDS) [20]: asymptomatic individual, (EDSS and OMDS—0 on both scales) and definite diagnosis of HAM (EDSS and OMDS greater than 1 on both scales).

Individuals with positive serology for the Human Immunodeficiency Virus (HIV), HTLV-2, or any other blood-tested disease were excluded, as well as an undetermined serology for HTLV-1 and a positive *Venereal Disease Research Laboratory* (VDRL) test.

Concerning all the participants, we excluded the individuals with neurological diseases, otitis, tympanic membrane perforation, history of otologic surgery or peripheral vestibular disease, as well as individuals with any alteration in the clinical neurological examination of the cranial nerves or unable to perform cervical rotation.

### Vestibular evoked myogenic potential (VEMP)

VEMP was performed with Labat^®^ equipment, using two channels. The stimuli were presented through ER 3A insertion phones, with disposable foam eartips. Tone burst stimuli at an intensity of 120 decibels normalized hearing level (dB nHL) were used. In this study, a bandpass filter of 10 to 1,500 Hertz (Hz) was used. To obtain each record, 100 stimuli were presented at a frequency of 500 Hz at a rate of four stimuli per second. The total duration of the 100 stimuli was 25 seconds. The scan window was 50 milliseconds (ms). Each subject underwent at least two stimulations per side, to verify the replication of the potential, and each stimulation was composed of 200 stimuli repeated in two consecutive cycles, no plateau (ramp = 2 and plateau = 0). The impedance values, which had to be below 5 kiloohm (KΩ), were checked before each record [11].

The recording of cervical VEMP and ocular VEMP was performed simultaneously. Channel 1 electrodes were used to record ocular VEMP and channel 2 electrodes to record cervical VEMP [11].

**Fig 1:**
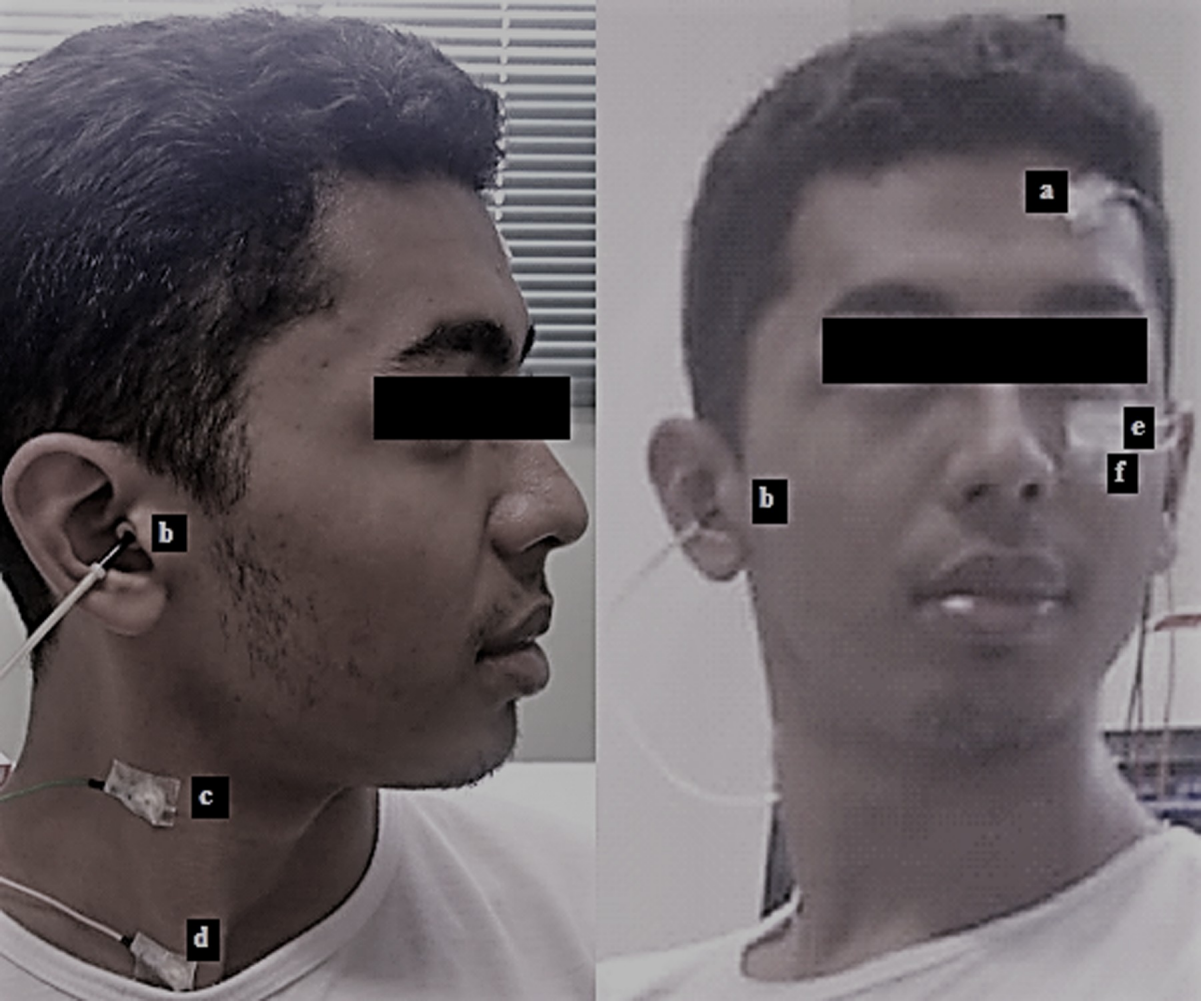
Simultaneous cervical and ocular VEMP. (a) ground electrode. (b) auditory stimulus. (c) active electrode on channel 2 at the anterior border of the sternocleidomastoid muscle in its upper third. (d) reference electrode on channel 2 at the sternal notch region. (e) active electrode on channel 1 below the lower eyelid. (f) reference electrode on channel 1 below the active electrode. The written informed consent was given by the person.

The active electrode related to cervical VEMP was placed on the same side of the auditory stimulus at the anterior border of the sternocleidomastoid muscle in its upper third, and the reference electrode was placed in the sternal notch region. For ocular VEMP recording, the active electrode (negative electrode) in channel 1 was placed approximately 1 centimeter (cm) below the lower eyelid of the opposite side of the auditory stimulus, and the reference electrode (positive electrode) was placed at a distance of approximately 1 cm from the active electrode. The ground electrode was placed on the forehead (Fpz) (Fig 1).

Participants were instructed to sit on the chair and keep their heads rotated to the opposite side of the stimulated ear, causing contraction of the sternocleidomastoid muscle. At the same time, the participant was instructed to look at a stationary target located on the wall in front of him and then immediately at a fixed point located above the target, which formed a vertical viewing angle of approximately 30° above the horizontal plane. The protocol of simultaneous ocular and cervical VEMP is available at doi.org/10.17504/protocols.io.zmzf476.

**Fig 2:**
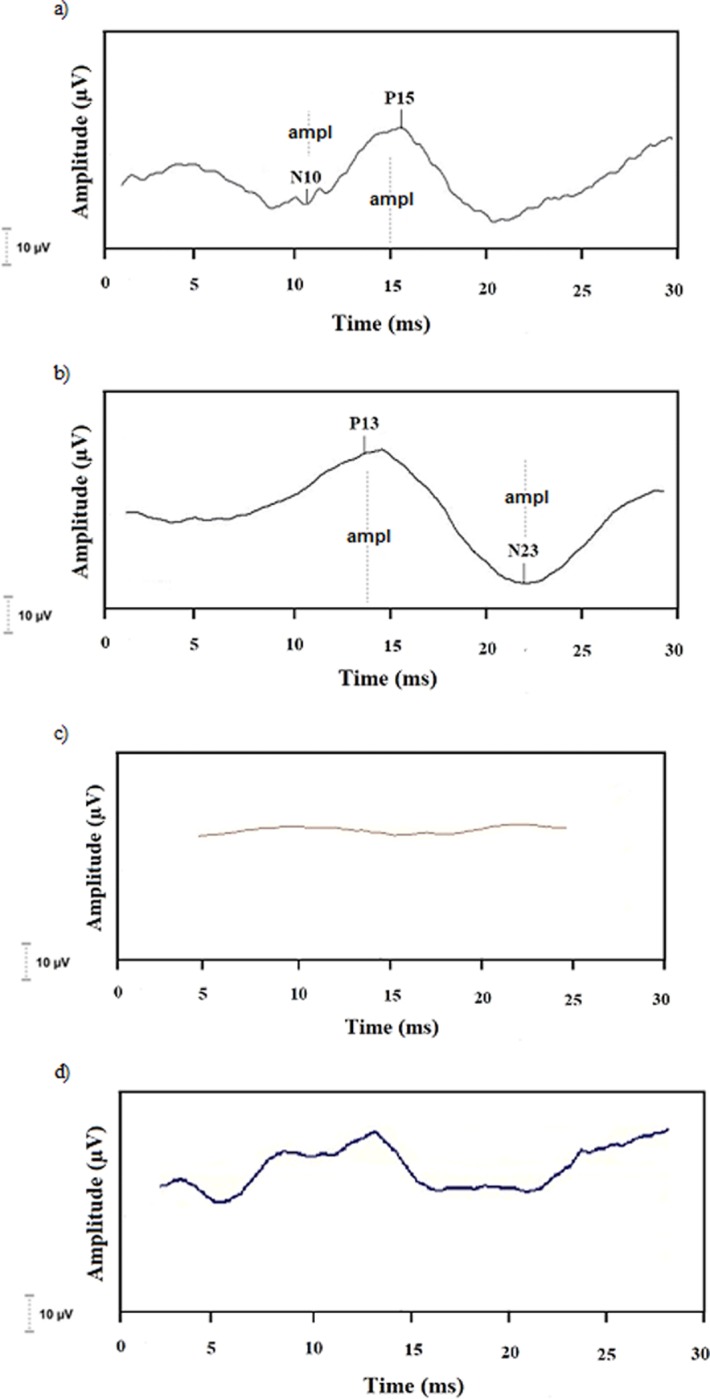
Examples of tracings obtained by the VEMP records. a) normal ocular VEMP. b) normal cervical VEMP. c) altered ocular VEMP (no response). d) altered cervical VEMP (no response).

The ocular VEMP is composed of two sets of biphasic waveforms. The first biphasic potential has a negative peak (N) with an average latency of 10 ms, followed by a positive peak (P) with an average latency of 15 ms, which is known as N10–P15. The cervical VEMP consists of two sets of biphasic waveforms. The first biphasic potential has a positive peak (P) with an average latency of 13 milliseconds (ms), followed by a negative peak (N) with an average latency of 23 ms, which it known as P13–N23 (Fig 2).

The American Society of Encephalography and Evoked Potentials’ criteria for evoked potentials were considered for the analysis of the latency values of the cervical VEMP and ocular VEMP waves. The definition of altered latency values includes those that exceed 2.5 standard deviations (SD) [21]. Thus, for ocular VEMP, we considered normal latency values within the range of 7.5 to 12.5 ms for N10 and within the range of 12.5 to 17.5 ms for P15. For cervical VEMP, we considered normal latency values within the range of 10.5 to 15.5 ms for P13 and within the range of 20.5 to 25.5 ms for N23 [22,23]. The validation of the analyzed reference values was guaranteed by comparing these with parameters already established in other national and international peer reviews [11,24,25].

The parameters considered in the VEMP analysis are the latency and amplitude of the waves. However, the amplitude may vary according to age, muscular strength [24,26], and Meniere's disease [27,28]. Therefore, amplitude was not considered in the analysis since this variable is not consistent to define neural conduction abnormalities.

VEMP results were classified as normal and altered. Latency prolongation and no response were considered as the altered results. Ocular and cervical VEMP were compared between the groups infected and not infected by HTLV-1.

### Statistical analysis of data

Statistical analysis was performed using the *Statistical Package for Social Sciences* (SPSS), version 20.0. Regarding the continuous variables (latencies in milliseconds) the normality of the samples was assessed using the Kolmogorov-Smirnov and Shapiro-Wilk tests. Since the distribution of the variables was not normal, the comparison between groups was performed using the Kruskal-Wallis test. Chi-square or Fisher's Exact test were used to compare nominal variables between groups. The adopted level of significance was 5% (p≤0.05). The magnitude of the difference of normal or altered VEMP between groups was calculated using the odds ratio (OR). The confidence interval (95%) was calculated in order to evaluate the sample variability.

## Results

The characteristics of the studied population and the classification in the neurological scales are described in Table 1. The groups were similar regarding gender and age.

**Table 1:** Comparison of the groups with HTLV-1-associated myelopathy (HAM), asymptomatic infection and healthy controls according to general characteristics and the disability scales (EDSS and OMDS).

Variables	Control (n = 26)	Asymptomatic (n = 26)	HAM (n = 26)	p-value
**Age**	53.27 (3.39)	53.73 (7.65)	55.69 (4.44)	0.073^a^
**Female** **Male**	18 [69.2] 8 [30.8]	16 [61.5] 10 [38.5]	19 [73.1] 7 [26.9]	0.662^b^
**EDSS**	0 (0)	0 (0)	3 (2.08)	≤0.001^a^
**OMDS**	0 (0)	0 (0)	2.30 (1.85)	≤0.001^a^

EDSS, expanded disability status scale; OMDS, Osame motor disability score; n, number of participants. Data are expressed as mean (standard deviation); absolute number [percentage].

^a^ Kruskal-Wallis Test (p≤0.05)

^b^ Chi-square Test (p≤0.05)

The VEMP latencies were different among the groups. Table 2 indicates the comparative analysis and identifies the groups with a relevant difference. The data points behind means, medians and variance measures was included in S2 Table.

**Table 2:** Comparison of the groups with HTLV-1-associated myelopathy, asymptomatic infection and healthy controls according to the latency (ms) of cervical VEMP (P13 and N23 waves) and ocular VEMP (N10 and P15 waves).

Variables		G1 (n = 26)	G2 (n = 26^a^)	G3 (n = 26^b^)	p - value *	Comparison groups	p - value **
Ocular VEMP	Lat N10	10.49 (0.65)	10.38 (0.92)	11.51 (2.80)	0.375	-	-
	Lat P15	15.40 (0.66)	15.74 (1.35)	18.17 (3.30)	≤0.001	G1 X G2 G1 X G3 G2 X G3	1.000 ≤0.001 0.002
Cervical VEMP	Lat P13	12.80 (0.91)	13.73 (1.03)	14.83 (3.22)	0.001	G1 X G2 G1 X G3 G2 X G3	0.007 0.002 1.000
	Lat N23	22.30 (1.36)	23.04 (2.44)	25.75 (4.43)	0.003	G1 X G2 G1 X G3 G2 X G3	0.919 0.003 0.060

G1, group control; G2, group of asymptomatic individuals; G3, group of individuals with HAM; SD, standard deviation; Lat, latency; n, number of participants. Data are expressed as mean (standard deviation).

^a^For Lat N23 data analysis, one case in which the response was absent was excluded.

^b^For Lat P13 and Lat N23 data analysis, 3 and 6 cases, respectively, in which the response was absent were excluded. For Lat N10 and Lat P15 data analysis, 2 and 3 cases, respectively, in which the response was absent were excluded.

* Kruskal-wallis Test (p≤0.05).

** Bonferroni Test.

Table 3 describes the frequency of normal and altered results for cervical and ocular VEMP in each group. The seronegative group of healthy controls was not included in the table because of normality in all the exams (cervical and ocular VEMP). The comparison of the controls with the group of asymptomatic carriers showed no difference between groups for ocular VEMP (p = 0.118) and difference for cervical VEMP (p = 0.002).

**Table 3:** Comparison of the groups with HTLV-1-associated myelopathy (HAM), asymptomatic infection and healthy controls according to the result (normal/altered) of ocular and cervical VEMP.

VEMP type	VEMP result						
	Comparison between groups	Normal - n (%)	Altered - n (%)		p-value	OR	CI (inferior-superior)
			Delayed wave	Absent wave			
**Ocular** **n (%)**	**Asymptomatic**	23 (88.5)	3 (11.5)		< 0.001	3.28	1.71–6.28
			3 (11.5)	0 (0.0)			
	**HAM**	7 (26.9)	19 (73.1)				
			8(30.8)	11(42.3)			
**Cervical** **n (%)**	**Asymptomatic**	18 (69.2)	8 (30.8)		< 0.001	6.00	2.00–17.93
			1(3.8)	7(26.9)			
	**HAM**	3 (11.5)	23 (88.5)				
			2(76.9)	21(80.8)			
**Cervical and Ocular n (%)**	**Asymptomatic**	25 (96.1)*	1 (3.8)**		< 0.001	2.5	1.52–4.090
	**HAM**	10 (38.5)*	16 (61.5)**				

n, number of participants; OR, odds ratio; CI, confidence interval; Data are expressed as absolute number (percentage).

*Normal cervical and ocular VEMP or just one altered.

**Cervical and Ocular VEMP altered.

Chi-square test or Fisher's Exact (p≤0.05)

**Fig 3:**
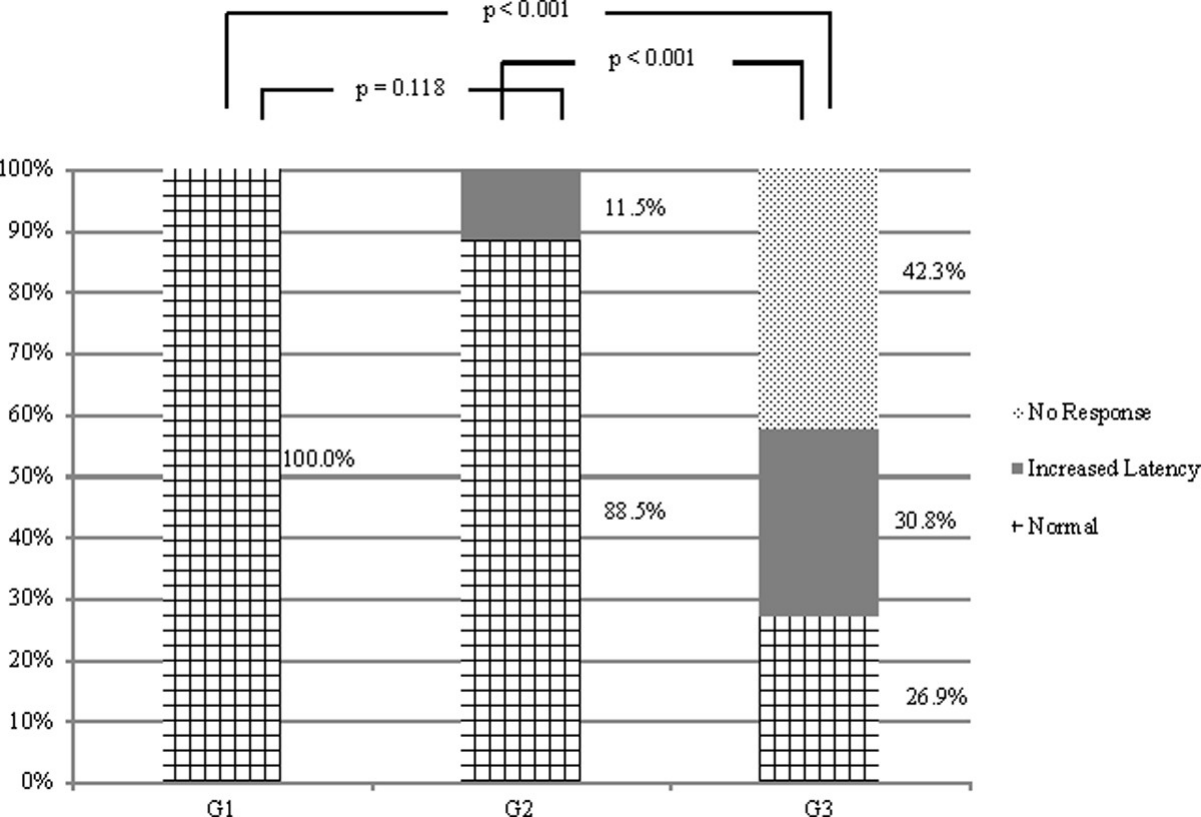
Comparison of ocular VEMP responses in individuals with HTLV-1-associated myelopathy, with asymptomatic infection and seronegative controls (n = 78). G1, group control; G2, group of asymptomatic individuals; G3, group of individuals with HAM. Chi-square or Fisher's Exact test (p≤0.05).

**Fig 4:**
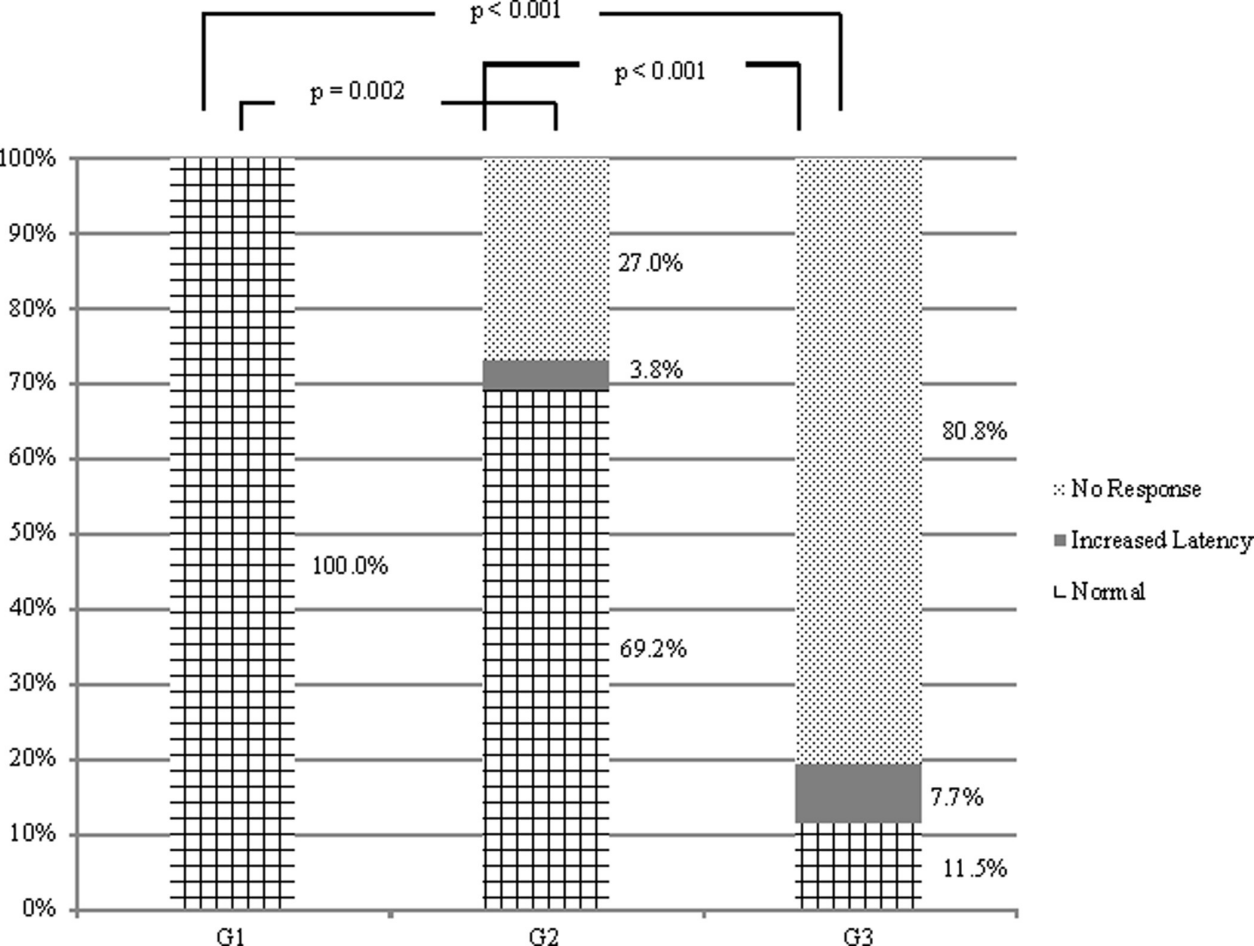
Comparison of cervical VEMP responses in individuals with HTLV-1-associated myelopathy, with asymptomatic infection and seronegative controls (n = 78). G1, group control; G2, group of asymptomatic individuals; G3, group of individuals with HAM. Chi-square or Fisher's Exact test (p≤0.05).

The VEMP result was categorized as 1) latency delay of N10-P15 waves (ocular) or of P13-N23 waves (cervical); 2) absence of wave; 3) normal wave. Fig 3 shows the comparative analysis for ocular VEMP and Fig 4 for cervical VEMP.

## Discussion

The auditory stimulus that evokes VEMP goes through the vestibular regions of the brain, especially the pre-motor cortex, the inferior and medial temporal gyrus, the Brodmann area, as well as the typically auditory areas, such as the primary auditory cortex [29].

The latency delay of cervical VEMP has been related to the demyelination of the primary afferent axon of the vestibulo-collic tract and/or involvement of the vestibular nucleus [30–32]. The absence of electrophysiological response may be explained by a severe impairment of the vestibular-spinal pathway [33].

When the evoked potential changes from a prolonged latency to no response, it is understood that there is a worsening in the neuronal damage [11,24,25]. This pattern of response was previously observed in a cohort study of individuals infected by HTLV-1 with myelopathy and asymptomatic carriers that were tested by cervical VEMP [8,13]. Axonal degeneration along the spinal cord may play a more prominent role in disease progression in HAM/TSP and may explain the significant correlation of VEMP alteration in early stage HAM and even in the HTLV-1-asymptomatic carrier who has more chance of evolving to HAM [13].

Regarding cervical VEMP in the present study, we found that the great majority of the patients with definite HAM presented alteration in cervical VEMP response (88,5%). This data confirms previous studies that disclosed a cervical spinal cord damage in HAM, emphasizing that the medullary abnormalities in HAM are not restricted to the thoracolumbar level [13,34,35].

Regarding ocular VEMP, 61.5% of the patients with definite HAM and alteration in cervical VEMP, presented also alteration in ocular VEMP (Table 3). The neural connections involved in the generation of ocular VEMP are mainly mesencephalic [19,36–38]. The presumed pathway includes the vestibular primary afferent, the vestibular nuclear complex, the medial longitudinal fasciculus, the oculomotor nucleus and the oculomotor nerves [36]. Thus, a latency delay or an absence of response depends on the disorganization of the primary afferents involved in the vestibulo-ocular reflex [36,37]. Currently, the major pathophysiological mechanism considered to explain CNS involvement in the HTLV-1 neurological disease is an immuno-mediated chronic inflammatory process in response to HTLV-1 infection, which damages nearby CNS components [39]. The higher frequency of simultaneous alteration in ocular and cervical VEMP in HAM group confirms a spread of neurological impairment in these individuals when compared to the group with asymptomatic infection.

VEMP can detect subclinical neurological changes in HTLV-1 infection [13,16,29]. We showed that many of those labeled as asymptomatic carriers, presented altered cervical VEMP and normal ocular VEMP, indicating alteration in cervical spine but not in the upper CNS. When effective therapeutic options for HTLV-1 neurological disease are available, the subclinical diagnosis of neuronal injury will have implications in decision-making regarding the beginning of the treatment in the stage of incipient damage. For example, recent studies have shown that low doses of corticosteroid can be beneficial in slowing HAM progression if treatment is implemented at the onset of the HTLV-1-neurological manifestation [40].

### Limitations

We do not include image and immunological data, such as proviral load and inflammatory markers in the serum. In the GIPH cohort, neither the image nor the proviral load have a good positive predictive value for the classification of the patients in the spectrum of HTLV-1 neurological disease [41,42].

The spinal cord atrophy seen in magnetic resonance imaging (MRI) and clinical disability have been correlated in advanced HAM [43], but not in early stage HAM [44]. The serum inflammatory profile that is typical of HAM does not present a good validity to be used in the clinical practice to differentiate the real HTLV-1-aymptomatic carrier from those who are developing HAM [45,46]. Therefore, although image and immunological data could add information to the present work, the lack of these results does not influence the valuable information that in HTLV-1 infection classified as asymptomatic, the cervical spine can present subclinical alterations, but not the upper CNS. On the contrary, in HAM, abnormal cervical VEMP and ocular VEMP have showed alterations in the spine as well as in the upper CNS, which is in line with the findings of cognitive alterations in HAM [47].

In HTLV-1-asymptomatic infection, subclinical alterations can be disclosed by electrophysiological tests but not by MRI [44].

## Conclusion

The mesencephalic impairment in HAM, showed by ocular VEMP changes, confirmed that HTLV-1 neurological disease in HAM is not restricted to the spinal cord. The alteration of the oculomotor system responsible for the eye stabilization during head and body movements can explain the high frequency of dizziness in patients with HAM. The impairment in cervical spine, showed by cervical VEMP changes, was found in HAM as well as in asymptomatic carriers. This fact shows that cervical spinal cord damage is indeed frequent and may represent an earlier stage in in relation to upper CNS involvement.

## Supporting information

S1 Appendix Questionnaire. (PDF)

S1 Data List Dataset of absolute values of the latencies according to sex, age, group of study and neurological scales. (XLSX)

S1 Table Diagnostic criteria of human T-cell lymphotropic virus type 1 (HTLV-1)- associated myelopathy (HAM)^a^. ^a^Castro-costa CMDE, Araújo AQC, Barreto MM, Takayanagui OM, Sohler MP, Silva ELMDA, et al. Proposal for diagnostic criteria of tropical spastic paraparesis/HTLV-1-associated myelopathy (HAM/TSP). AIDS Res Hum Retroviruses. 2006;22:931–935. Doi: 10.1089/aid.2006.22.931.
(PDF)

S2 Table Descriptive variables of healthy controls, asymptomatic infection group, and HTLV-1-associated myelopathy group: Age, disability scales (EDSS and OMDS), the latency (ms) of cervical VEMP (P13 and N23 waves) and ocular VEMP (N10 and P15 waves). (PDF)
