# Supplementary material for: Ocular vestibular evoked myogenic potential (VEMP) reveals mesencephalic HTLV-1-associated neurological disease
Source: PLoS One. 2019 Dec 27;14(12):e0217327. doi: 10.1371/journal.pone.0217327 (PMC6934290; doi:10.1371/journal.pone.0217327)
Supplement: S1 Appendix — (PDF) [file pone.0217327.s001.pdf]

QUESTIONNAIRE

IDENTIFICATION

ID _________ IDSEARCH _________ GIPH ____________ DATE OF EXAMINATION ___/___/_____

NAME ____ SEX ______________________

ADDRESS ____

TELEPHONE ______________ / ______________ E-MAIL ______________________________

DATE OF BIRTH _____/_____/_____ AGE _________ SCHOOLING YEARS _________

HOURS OF SLEEP IN THE PREVIOUS NIGHT ________ DOMINANT HAND ______ GROUP _______

GENERAL HEALTH

MEDICATION _____ WHICH ____

HEARING COMPLAINT _____ WHICH______________________________ TINNITUS _____

VESTIBULAR COMPLAINT _____ WHICH______________________________ CONVULSION ________________

MEMORY COMPLAINT _________ COMPLAINT ATTENTION ________ COMPLAINT COMMUNICATION ________

NEUROLOGICAL EXAMINATION

WORSENING/IMPROVEMENT _____ DESCRIBE ____________________________________

NEW EXAMINATION SURVEY _____ REALIZED ______ RESULT ______ CURRENT CLASSIFICATION __
