# Supplementary material for: Ocular vestibular evoked myogenic potential (VEMP) reveals mesencephalic HTLV-1-associated neurological disease
Source: PLoS One. 2019 Dec 27;14(12):e0217327. doi: 10.1371/journal.pone.0217327 (PMC6934290; doi:10.1371/journal.pone.0217327)
Supplement: S1 Table — aCastro-costa CMDE, Araújo AQC, Barreto MM, Takayanagui OM, Sohler MP, Silva ELMDA, et al. Proposal for diagnostic criteria of tropical spastic paraparesis/HTLV-1-associated myelopathy (HAM/TSP). AIDS Res Hum Retroviruses. 2006;22:931–935. Doi: 10.1089/aid.2006.22.931. (PDF) [file pone.0217327.s003.pdf]

S1 Table. Diagnostic criteria of human T-cell lymphotropic virus type 1 (HTLV-1)- associated myelopathy (HAM)

**Definite:**<br>1. A non-remitting progressive spastic paraparesis with sufficiently impaired gait to be perceived by the patient. Sensory symptoms or signs may or may not be present. When present, they remain subtle and without a clear-cut sensory level. Urinary and anal sphincter signs or symptoms may or may not be present.<br>2. Presence of HTLV-1 antibodies in serum and cerebrospinal fluid (CSF) confirmed by Western blot and/or a positive real-time polymerase chain reaction (PCR) for HTLV-1 in blood and/or CSF.<br>3. Exclusion of other disorders that can resemble HAM.
**Probable:**<br>1. Mono-symptomatic presentation: spasticity or hyperreflexia in the lower limbs or isolated Babinski sign with or without subtle sensory signs or symptoms, or neurogenic bladder only confirmed by urodynamic tests.<br>2. Presence of HTLV-1 antibodies in serum and/or CSF confirmed by Western blot and/or a positive PCR for HTLV-1 in blood and/or CSF.<br>3. Exclusion of other disorders that can resemble HAM.
**Possible:**<br>1. Complete or incomplete clinical presentation.<br>2. Presence of HTLV-1 antibodies in serum and/or CSF confirmed by Western blot and/or a positive PCR for HTLV-1 inblood and/or CSF.<br>3. Disorders that can resemble HAM have not been excluded.
