# Supplementary material for: Ocular vestibular evoked myogenic potential (VEMP) reveals mesencephalic HTLV-1-associated neurological disease
Source: PLoS One. 2019 Dec 27;14(12):e0217327. doi: 10.1371/journal.pone.0217327 (PMC6934290; doi:10.1371/journal.pone.0217327)
Supplement: S2 Table — (PDF) [file pone.0217327.s004.pdf]

S2 Table. Descriptive variables of healthy controls, asymptomatic infection group, and HTLV-1-associated myelopathy group: age, disability scales (EDSS and OMDS), the latency (ms) of cervical VEMP (P13 and N23 waves) and ocular VEMP (N10 and P15 waves).

Variables		Control (n=26)	Asymptomatic (n=26)	HAM (n=26)
**Age**	Mean	53.27	53.73	55,69
	Standard deviation	3.39	7.65	4.44
	Median	53.00	58.50	57.00
	Minimum	46.00	32.00	45.00
	Maximum	59.00	60.00	60.00
**EDSS**	Mean	0.00	0.00	3.00
	Standard deviation	0.00	0.00	2.08
	Median	0.00	0.00	2.00
	Minimum	0.00	0.00	1.00
	Maximum	0.00	0.00	7.00
**OMDS**	Mean	0.00	0.00	2.30
	Standard deviation	0.00	0.00	1.85
	Median	0.00	0.00	1.00
	Minimum	0.00	0.00	1.00
	Maximum	0.00	0.00	6.00
**Cervical VEMP P13 latency**	Mean	12.80	13.73	14.83
	Standard deviation	0.91	1.03	3.22
	Median	12.73	13.85	13.85
	Minimum	11.15	11.25	12.00
	Maximum	14.70	16.40	23.00
**Cervical VEMP N23 latency**	Mean	22.30	23.04	25.75
	Standard deviation	1.36	2.44	4.43
	Median	21.88	22.63	24.63
	Minimum	20.50	20.50	20.50
	Maximum	24.80	33.00	33.00
**Ocular VEMP N10 latency**	Mean	10.49	10.38	11.51
	Standard deviation	0.65	0.92	2.80
	Median	10.70	10.33	10.38
	Minimum	8.75	9.00	9.05
	Maximum	11.45	13.55	20.00
**Ocular VEMP P15 latency**	Mean	15.40	15.74	18.17
	Standard deviation	0.66	1.35	3.30
	Median	15.44	15.68	16.85
	Minimum	14.10	13.75	14.20
	Maximum	16.60	20.90	25.00
